# Bridging the Gap: Type III Secretion Systems in Plant-Beneficial Bacteria

**DOI:** 10.3390/microorganisms10010187

**Published:** 2022-01-15

**Authors:** Antoine Zboralski, Adrien Biessy, Martin Filion

**Affiliations:** Research and Development Centre, Agriculture and Agri-Food Canada, 430 Gouin Boulevard, Saint-Jean-sur-Richelieu, QC J3B 3E6, Canada; antoine.zboralski@agr.gc.ca (A.Z.); adrien.biessy@agr.gc.ca (A.B.)

**Keywords:** type III secretion system, rhizobia, *Pseudomonas*, rhizosphere, PGPR, biocontrol, nodulation, mycorrhization, plant immunity, protists

## Abstract

Type III secretion systems (T3SSs) are bacterial membrane-embedded nanomachines translocating effector proteins into the cytoplasm of eukaryotic cells. They have been intensively studied for their important roles in animal and plant bacterial diseases. Over the past two decades, genome sequencing has unveiled their ubiquitous distribution in many taxa of Gram-negative bacteria, including plant-beneficial ones. Here, we discuss the distribution and functions of the T3SS in two agronomically important bacterial groups: the symbiotic nodule-forming nitrogen-fixing rhizobia and the free-living plant-beneficial *Pseudomonas* spp. In legume-rhizobia symbiosis, T3SSs and their cognate effectors play important roles, including the modulation of the plant immune response and the initiation of the nodulation process in some cases. In plant-beneficial *Pseudomonas* spp., the roles of T3SSs are not fully understood, but pertain to plant immunity suppression, biocontrol against eukaryotic plant pathogens, mycorrhization facilitation, and possibly resistance against protist predation. The diversity of T3SSs in plant-beneficial bacteria points to their important roles in multifarious interkingdom interactions in the rhizosphere. We argue that the gap in research on T3SSs in plant-beneficial bacteria must be bridged to better understand bacteria/eukaryotes rhizosphere interactions and to support the development of efficient plant-growth promoting microbial inoculants.

## 1. Introduction

Type III secretion systems (T3SSs) are syringe-like membrane-embedded nanomachines that enable the translocation of effector proteins directly into the cytoplasm of eukaryotic cells. The T3SS injectosome is made of more than 20 proteins and is evolutionary related to the bacterial flagellum [1,2]. It is widely distributed among Gram-negative bacteria [3]. The T3SS has historically been known as a major virulence determinant in many important human pathogens, especially those belonging to the *Yersinia*, *Salmonella* and *Chlamydia* genera [4,5,6]. It is also a major plant pathogenicity factor in several bacterial taxa [7]. For instance, the plant pathogenic bacterium *Pseudomonas syringae* relies on its T3SS to translocate effector proteins into plant cells to modulate the host defense responses and promote infection [8].

Even though T3SSs are used by pathogenic bacteria to promote infection and manipulate the host immune system, they can be found in many non-pathogenic bacteria. Hu et al. performed a comprehensive survey of about 20,000 available bacterial genomes to search for T3SS gene clusters [3]. These were found in 109 genera, including many environmental strains with no known association with eukaryotic hosts, but also plant-beneficial bacteria, such as rhizobia and *Pseudomonas* isolates.

Rhizobia are a paraphyletic group that includes the *Rhizobium*, *Bradyrhizobium*, *Sinorhizobium* (*Ensifer*), and *Mesorhizobium* genera. They are known for their symbiotic relationships with numerous legumes, mediated by nitrogen-fixing nodules sheltering the bacteroids. The development of these symbiotic organs requires a complex coordination between rhizobial infection and nodule organogenesis, bringing into play the secretion and recognition of signal molecules by both partners [9]. To facilitate the rhizobia-legume symbiosis, numerous rhizobial strains carry one or multiple T3SS biosynthetic gene clusters [10,11,12,13], as well as genes encoding effector proteins secreted through the T3SS [14].

T3SSs are also found in free-living plant-beneficial bacteria belonging to the genus *Pseudomonas* [15,16,17]. These bacteria aggressively colonize the rhizosphere and promote plant growth, either directly by producing plant hormones and solubilizing phosphate, or indirectly by suppressing soil-borne diseases [18,19]. Contrary to the T3SSs found in rhizobia, T3SSs in plant-beneficial *Pseudomonas* spp. seem to have other functions than the sole interaction with the plant.

In this perspective paper, we discuss the distribution and functions of T3SSs and their effectors in plant-beneficial bacteria. We specifically focus on two agronomically important bacterial groups, namely the rhizobia and the plant-beneficial *Pseudomonas* spp., to highlight the gap in T3SS research on plant-beneficial bacteria and the potential benefits of bridging this gap.

## 2. T3SSs in Rhizobia-Legume Interaction: Symbiotic Determinants with Multiple Roles

Numerous nitrogen-fixing rhizobia carry a functional T3SS [10,11,12,13]. The T3SS gene clusters found in the genomes of rhizobia belong to the Rhizobiales family (Rhc-T3SS) [20,21,22]. This family can be divided into four subgroups, namely α-RhcI, α-RhcII, α-RhcIII, and β-Rhc. Most rhizobia harbor a α-RhcI T3SS [22], but some strains harbor an additional T3SS gene cluster belonging to the α-RhcII subgroup of the Rhizobiales-T3SS family [23,24]. Interestingly, this T3SS gene cluster can also be found in several plant-pathogenic *P*. *syringae* strains [21]. The role of this second T3SS remains to be characterized.

Nitrogen-fixing rhizobia use their T3SS to secrete effectors (designated as nodulation outer proteins, Nops) into the cytoplasm of legume cells [14]. The effector repertoire of numerous rhizobial strains has been characterized [14,22,25]. For example, Teulet et al. studied the distribution of known Nop effectors in the genome of 92 T3SS-harboring *Bradyrhizobium*. The *Bradyrhizobium* strains harboring a RhcI-T3SS gene cluster display two to twenty-four effector-encoding genes [22]. Interestingly, not a single effector is shared by all T3SS-carrying *Bradyrhizobium* strains, indicating the absence of a core effectome. Nevertheless, four effectors (NopM, NopP, NopT, and NopC) are conserved in most *Bradyrhizobium* strains with a RhcI-T3SS and in other rhizobial genera as well [22]. Effectors secreted by rhizobial strains have various functions, including the promotion of bacterial infection by modulating the plant immune response and the initiation of nodule organogenesis.

### 2.1. Modulating the Plant Immune Response

Several studies have shown that the inoculation of rhizobia in the vicinity of leguminous root cells induces a transient increase in the expression of numerous plant defense-related genes [26,27,28], corresponding to the microbe-associated molecular pattern (MAMP)-triggered immunity. The basis of this MAMP-triggered immunity remains poorly characterized, as no rhizobial MAMP has been shown to be active on legumes [29]. The defense reaction is rapidly suppressed by the rhizobia, thanks to the action of nodulation factors and exopolysaccharides [29,30,31]. In addition, rhizobial symbionts employ T3SS effectors to modulate the plant immune response and to suppress the MAMP-triggered immunity. For example, the effector NopL found in the *Sinorhizobium fredii* strains NGR234 and HH103, as well as in *B. elkanii* USDA61 [32,33,34], is translocated into legume cells. It represses the expression of several genes encoding pathogenesis-related defense proteins associated with MAMP-triggered immunity, preventing nodule senescence [32]. This effector is phosphorylated by mitogen-activated plant kinases (MAPK) and interfere with MAPK signaling [35,36], thus weakening the plant immune response. Another effector, NopM, is also secreted by rhizobial symbionts to modulate legume defense responses. This effector contains a Novel E3 ubiquitin ligase (NEL) domain and its expression in *Nicotiana benthamiana* strongly reduced the generation of reactive oxygen species (ROS), one of the main MAMP-triggered immunity responses [37]. NopM belongs to a large family of bacterial effectors that manipulate the plant ubiquitin systems [38,39]. It forms unanchored polyubiquitin chains in vivo, potentially targeting plant defense proteins for proteasome-dependent degradation [40].

### 2.2. Initiating the Nodulation Process in the Absence of Nodule Factor Signaling

Nitrogen-fixing bacteria detect flavonoid molecules exuded by legume roots and secrete lipo-chitooligosaccharide signal molecules called nodulation factors (NFs). NFs are recognized by plant membrane-embedded receptors with extracellular LysM domains (NFRs) [41,42]. This recognition of NFs by NFRs triggers various host responses, leading to nodule organogenesis. Several rhizobial strains were shown, however, to nodulate some legume species in the absence of NF signaling. For example, *B*. *elkanii* USDA61 can activate nodulation signaling and nodule formation in a *nfr1* soybean mutant lacking functional NF receptors [43]. Similarly, a USDA61 mutant impaired in NF production was still able to nodulate soybean. On the other hand, USDA61 T3SS-knockout mutants were unable to nodulate *nfr1* soybean mutants, suggesting that this rhizobial strain can nodulate soybean in a NF-independent but T3SS-dependent manner [43]. In addition, numerous *Bradyrhizobium* species are able to nodulate several legume species belonging to the *Aeschynomene* genus in a T3SS-dependent manner, but independently of NF signaling [44]. It was hypothesized that these microorganisms likely harbor effectors capable of inducing nodule organogenesis in the absence of NF signaling. An effector, called Bel2-5, was precisely shown to be crucial for nodule formation, as a *bel2-5* mutant was unable to nodulate *nfr1* soybean mutants [45]. This effector is found in several rhizobial genera, including *Bradyrhizobium*, *Sinorhizobium* and *Mezorhizobium*. Interestingly, Bel2-5 is not considered to be rhizobia-specific, as it shares similarities with XopD, an effector secreted by the plant pathogen *Xanthomonas campestris* [45]. Using an adenylate cyclase reporter system, this effector was confirmed to be translocated inside soybean cells [45]. Together with other effectors, such as NopL and ErnA, Bel2-5 likely modulates cytokinin biosynthesis-related genes involved in nodule organogenesis but also represses host-defense responses that could be detrimental to rhizobial infection.

### 2.3. Nops Elicit Effector-Triggered Immunity in some Legume Genotypes

In several cases, carrying a functional T3SS gene cluster or secreting a specific Nop effector protein has been shown to be detrimental to the nodulation of some legume species or genotypes [46,47,48]. For example, the effector protein NopT produced by *Rhizobium* sp. NGR234 promotes nodule formation in two legume species, *Phaseolus vulgaris* and *Tephrosia vogelii*, but is detrimental for the nodulation of another legume species, *Crotalaria juncea* [47]. Indeed, a NopT-deficient NGR234 mutant induces the formation of more nodules in *Crotalaria juncea* than the wild-type strain. In addition, there are nodulation restrictions between some legume species (or genotypes) and some rhizobial strains that completely prevent nodulation [49]. The basis of these nodulation restrictions has been extensively studied in soybean, which harbors various resistance genes involved in symbiosis incompatibility. For example, the *rj2/rfg1* gene encodes a Toll-interleukin receptor/nucleotide-binding site/leucine-rich repeat (TIR-NBS-LRR) protein that restricts nodulation by some strains of *B*. *japonicum* and *S*. *fredii* [50]. Other soybean cultivars harbor the *rj4* gene, which encodes a thaumatin-like protein belonging to the pathogenesis-related protein family 5 [51,52]. The rhizobial T3SS was shown to mediates the symbiotic incompatibility between *Rj2*/*Rfg1*/*Rj4* soybean genotypes and specific rhizobial strains [53,54]. These resistance proteins recognize effectors secreted by rhizobial T3SS. For example, the protein Rj4 recognizes Bel2-5 secreted by *B*. *elkanii* [54] and Rj2 recognizes the effector NopP produced by *B*. *diazoefficiens* USDA 122 [55]. The recognition of rhizobial T3SS effectors triggers plant defense responses similar to the SA-mediated effector-triggered immunity [49,54,56].

Rhizobial T3SSs and the effectors they translocate are important for symbiotic relationships with legumes. However, the functions of some rhizobial effectors and T3SSs remain unknown. Their characterization could help better understand how rhizobia interact with legumes, and maybe expand the host range of these symbionts.

## 3. T3SSs in Plant-Beneficial *Pseudomonas* Strains: Many Roles and Little Knowledge

**Figure 1:**
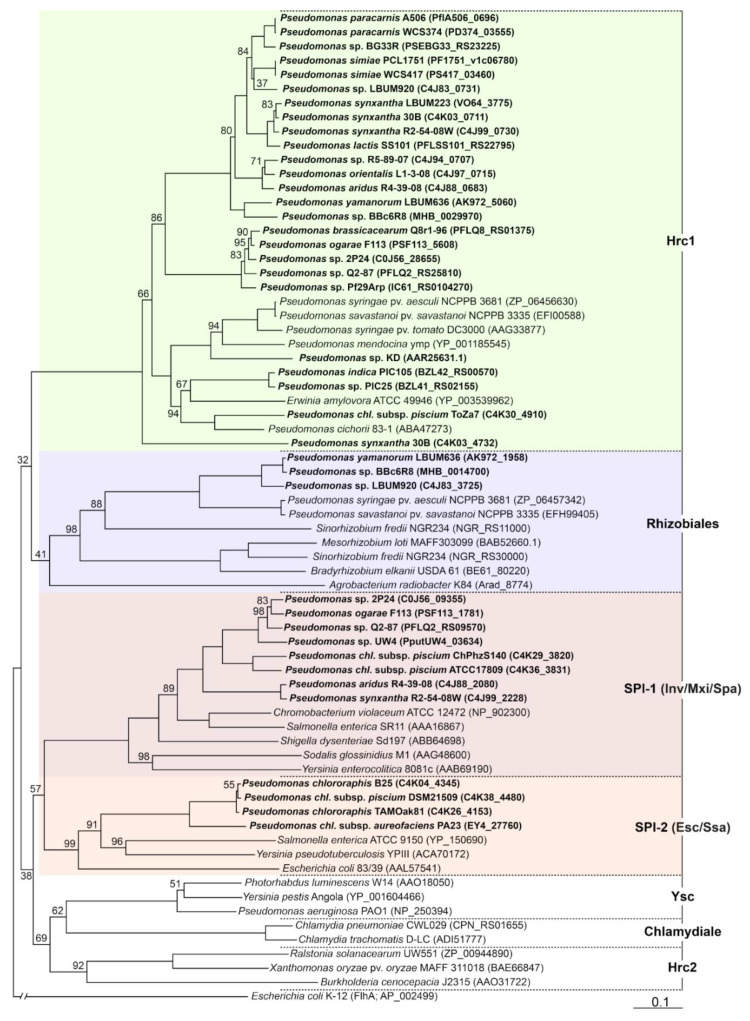
Neighbor-joining phylogeny of non-flagellar type III secretion systems based on the amino acid sequence of the major export apparatus protein SctV. The amino acid sequences were aligned using MUSCLE [80] and the tree was generated using the Geneious tree builder (Biomatters, Auckland, New Zealand) and the Jukes-Cantor method. The flagellar protein FlhA from *Escherichia coli* strain K12 was used as an outgroup. Bootstrap values different from 100% (out of 1000 replicates) are indicated at the nodes. T3SSs from plant-beneficial *Pseudomonas* spp. are highlighted in bold. The GenBank accession numbers are provided in brackets (*chl*., *chlororaphis*).

Next generation sequencing technologies have made the exploration of genomes of plant-beneficial *Pseudomonas* strains easier, enabling the discovery of T3SS gene clusters in these bacteria. Over the last two decades, complete T3SS clusters have been identified in at least 55 plant-beneficial *Pseudomonas* strains (Table 1). They have been assigned to four T3SS families, namely Hrc1 (Hrp1), Rhizobiales (Hrp3), SPI-1 (Inv/Mxi/Spa), and SPI-2 (Esc/Ssa), that are phylogenetically distinct (Figure 1) [15,17,20]. The Hrc1 family is the most common T3SS family found in plant-beneficial *Pseudomonas* spp. On the other hand, in *Pseudomonas* spp., the SPI-2 family is only found in a few strains of *P*. *chlororaphis*. Fifteen plant-beneficial *Pseudomonas* strains carry two complete T3SS gene clusters from distinct families. Interestingly, some of these *Pseudomonas* strains harbor a T3SS belonging to the Rhizobiales family. Many other strains have been described as carrying T3SS genes such as *sctRST* or *sctN*, but the completeness of the clusters was not investigated [57,58]. The list of T3SS-carrying phytobeneficial *Pseudomonas* strains may then be much longer. Here, we discuss the roles of these T3SSs and their associated effectors.

### 3.1. Manipulating the Plant Immune Response

In 2011, Mavrodi et al. demonstrated for the first time the active role played by a T3SS and its effectors in a rhizosphere-dwelling non-pathogenic *Pseudomonas* strain suppressing the plant immune response [61]. Three identified effectors in *P*. *brassicacearum* Q8r1-96, designated RopAA, RopB, and RopM, are directly secreted into plant cells (*Nicotiana tabacum*) through a T3SS. They can suppress the hypersensitive response and the oxidative burst, two immune responses related to the effector-triggered immunity and the MAMP-triggered immunity, respectively. Interestingly, the Q8r1-96 strain has also been shown to trigger induced systemic resistance (ISR) in *Arabidopsis thaliana* against *P*. *syringae*, using the polyketide 2,4-diacetylphloroglucinol [78]. These apparently contradictory effects on the immune system rather reflect the finely tuned interkingdom interactions necessary to establish mutually beneficial effects.

In a transcriptomic study on *A*. *thaliana* roots inoculated with a plant-beneficial *Pseudomonas* strain, *P*. *simiae* WCS417, Stringlis et al. showed that the bacterium was able to actively suppress a part of the MAMP-triggered gene expression response [79]. In another study, the same research team investigated the genome of this strain in search of T3SS gene clusters and effectors [16]. They found a unique T3SS cluster as well as a gene encoding RopE, an effector belonging to the AvrE family, known to suppress the plant immune response. They also identified 11 putative effectors, for the most part unrelated to known effector families. These effectors may be responsible for the impact of WCS417 on the plant immune system. Other plant-beneficial *Pseudomonas* strains have been successfully analyzed to identify T3SS putative effectors [15,16,62]. For some strains, up to 15 putative effectors have been found [15]. They may be involved in interactions with other eukaryotes than plants, such as mycorrhizal fungi, filamentous plant pathogens, or protists.

### 3.2. T3SSs in Mycorrhiza Helper Pseudomonas Strains: Who Is the Target?

Mycorrhizal symbioses can be affected by T3SSs carried by plant-beneficial *Pseudomonas* strains as well. This has first been demonstrated by Cusano et al., who knocked out several T3SS genes in *Pseudomonas* sp. BBc6R8, a known mycorrhiza helper bacterium [76]. The mutant was unable to promote colonization of Douglas fir by the fungus *Laccaria bicolor*, indicating the importance of the targeted T3SS in the symbiotic relationship. Similar findings were later reported by Viollet et al. with the *P*. *fluorescens* C7R12/*Funneliformis mosseae* BEG12/*Medicago truncatula* tripartite interaction [81]. In both studies, whether the T3SS impacted the fungus itself or the plant remains unclear, and the effectors involved in these proven T3SS-mediated interactions were not discovered. But in another study, Viollet et al. showed that T3SS-carriyng *Pseudomonas* isolates were enriched in the mycorrhizosphere of *M*. *truncatula*, suggesting a significant, broader role for these bacteria and for T3SSs in the complex symbiotic and mutualistic interactions taking place in the rhizosphere [82]. Exploring these roles further could help improve the use of these bacteria in the field, as they are already known for their biocontrol and/or mycorrhiza-beneficial abilities [83,84].

### 3.3. T3SSs as Subtle Weapons in the Battle against Plant Pathogens

T3SSs can also be involved in biocontrol activities of plant-beneficial *Pseudomonas* strains. This has been demonstrated by Rezzonico et al. using *Pseudomonas* sp. KD, an efficient biocontrol strain against the oomycete *Pythium ultimum* [68]. A mutation in *hrcV*, a gene encoding a T3SS structural protein, resulted in a strong reduction of the biocontrol activity against the oomycete on cucumber. The authors also showed that T3SS genes were upregulated in the presence of the oomycete and not in the presence of the plant, and that the bacterium was able to actively reduce the production of key virulence factors by *P*. *ultimum* without altering the oomycete growth in vitro. These results show that this bacterial strain directly affects *P*. *ultimum* by using its T3SS. The effectors involved in this interaction and their mode of action remain to be deciphered. To our knowledge, this is the first and only example of a T3SS-mediated biocontrol mechanism described in a plant-beneficial bacterium against a phytopathogenic oomycete.

Another study pinpointed the potential role of the T3SS in biocontrol by a *Pseudomonas* strain, *Pseudomonas* sp. Pf29Arp, this time against take-all disease in wheat, caused by the fungus *Gaeumannomyces tritici* [69]. Marchi et al. found that the *rscN* gene, which encodes a T3SS structural protein, was more expressed when the bacterium was growing on roots infected with the pathogenic fungus than when it was growing on healthy roots. In a previous study, they also showed that the amount of *G*. *tritici* mycelium was not affected by the bacterial strain, even though take-all symptoms were reduced. This points to a more subtle biocontrol mechanism than direct inhibition of the fungus through antibiosis, fungistasis or niche competition. They hypothesized that the T3SS may mediate interactions with the pathogenic fungus, with the host plant, or with both, leading to reduced symptoms.

More recently, Almario et al. have identified positive associations between the presence of a T3SS-encoding gene in multiple plant-beneficial *Pseudomonas* strains and their biocontrol abilities against *P*. *ultimum* and *Fusarium oxysporum* [85]. For example, more than 80% of the *Pseudomonas* isolates carrying the T3SS gene *hrcN* in this study displayed interesting in planta biocontrol levels against *P*. *ultimum*, while less than 25% of T3SS-lacking isolates did [85]. Non-T3SS genes positively associated with biocontrol effects were also identified in this study and may actively contribute to the conferred protection. Nevertheless, the presence of a T3SS gene cluster (or several) in these efficient biocontrol strains suggest a significant role of T3SSs in the interactions between *Pseudomonas* spp. and their eukaryotic neighbors, such as the previously mentioned T3SS-mediated role in *Pseudomonas* sp. KD/*P*. *ultimum* biocontrol interaction.

### 3.4. Being Eaten by Protists? No Thanks

Protists are mostly unicellular eukaryotes, including organisms that actively consume bacteria in soils [86]. Even though they are often neglected in rhizosphere research, they play an essential role as predators [87]. To counter protist predation, several human-pathogenic bacteria have been shown to use T3SSs, including bacteria from the *Pseudomonas* genus [88]. However, this has barely been studied in plant-beneficial *Pseudomonas* strains. To our knowledge, only Barret et al. have investigated the role played by a T3SS in such *Pseudomonas*-protist interactions [73]. They showed that the SPI-1 T3SS carried by “*P*. *ogarae*” F113—also known as *P*. *fluorescens* F113 and *P*. *kilonensis* F113 [89]—might be involved in resistance to predation by the amoeba *Acanthamoeba polyphaga* in mixed bacterial populations. Their results showed that the proximity of the amoeba increased the expression of *hilA*, a gene encoding a T3SS transcriptional activator in F113. Interestingly, Mavrodi et al. found a hemolysin-like gene in the strain Q8r1-96 that is probably activated by a T3SS regulation protein [61]. Hemolysins are toxins forming transmembrane pores, leading to cell lysis. The research team hypothesized that this hemolysin-like protein may improve the resistance abilities of this bacterium against bacteriovore predation. Further research is clearly needed to better understand the roles and mechanisms of T3SSs in the interactions between plant-beneficial *Pseudomonas* spp. and protists.

### 3.5. Are T3SSs Involved in Rhizocompetence?

Rhizocompetence is an essential trait for biocontrol activities of plant-beneficial *Pseudomonas* spp., relying on multiple determinants [90]. The contribution of T3SSs to this trait has been investigated using a reverse genetics approach in at least two *Pseudomonas* strains: *Pseudomonas* sp. KD and *P*. *brassicacearum* Q8r1-96 [61,68]. In the KD strain, Rezzonico et al. impaired the *hrcV* gene, encoding the T3SS major export apparatus protein [91]. The rhizosphere colonization ability of this mutant was assessed seven days after inoculation of pregerminated cucumber seeds grown in potting mix. The *hrcV* mutant colonized the rhizosphere of cucumber to the same extent as the wild type. In the Q8r1-96 strain, Mavrodi et al. impaired multiple T3SS structural protein genes, generating mutants lacking a functional T3SS. Long-term rhizosphere colonization was assessed in wheat and pea plants in soil, with each mutant alone or in competition with the wild type, over four successive 2-week growing cycles. Like the KD *hrcV* mutant, none of the Q8r1-96 mutants were affected in colonization. These results suggest that T3SSs are probably not involved in rhizocompetence. More recently, we assessed the rhizocompetence of 60 *Pseudomonas* strains, including T3SS carriers, in *A*. *thaliana* and potato [92]. We found that most of the best rhizosphere colonizers did not carry any complete T3SS gene cluster [17]. We also showed that T3SS carriers tended to colonize the rhizosphere of both plants to a lesser extent that strains not harboring any, suggesting that T3SS might potentially be detrimental to rhizocompetence. This remains to be further explored. However, as highlighted by Mavrodi et al. the T3SS might play a positive role earlier in the rhizosphere colonization process, especially at the root tip, given its proven role in the suppression of the plant immune response [61]. The potential role played by T3SSs in early rhizosphere colonization will have to be further investigated.

## 4. A Big Cog in the Nanomachine: Regulation of T3SSs

The transcriptional regulation of genes encoding the T3SS structural and effector proteins has been extensively studied in pathogenic strains from genera such as *Yersinia*, *Salmonella* and *Pseudomonas* [93]. In these bacteria, T3SS activity and gene expression are under the control of a central transcriptional activator, usually belonging to the AraC/XylS family, integrating both environmental and intracellular signals. This regulation takes place at multiple levels, especially involving RNA-binding proteins, regulatory RNAs, secreted effectors, and anti-sigma factors. They form a complex regulatory system that often varies from a strain or a species to another [93,94]. In plant-beneficial bacteria, these T3SS regulatory pathways have been much less investigated.

In several rhizobia species, the expression of T3SS genes is controlled by the DNA-binding protein TtsI, which is regulated by the flavonoid-dependent LysR-type regulator NodD1 [95]. TtsI binds to a conserved cis element (*tts* box), which is found in the promoter region of the T3SS structural and effector genes, activating the transcription of these genes. Given the complexity and diversity of T3SS regulatory networks in pathogens and the importance of T3SSs for rhizobia, there might be other regulatory mechanisms in rhizobia allowing a fine-tuning of the T3SS-mediated symbiosis with plants, which remain to be deciphered.

In plant-beneficial *Pseudomonas* strains, much less is known about T3SS regulation than in rhizobia. Using a T3SS gene promoter coupled to a luciferase operon, Mavrodi et al. have shown that T3SS genes were expressed by *P*. *brassicacearum* Q8r1-96 in the rhizosphere of wheat grown in soil, validating gene expression previously observed in vitro in another plant-beneficial *Pseudomonas* strain [61,96]. In several plant-beneficial *Pseudomonas* spp., a conserved motif, called *hrp* box, has been found in T3SS gene promoters [64,69,73]. In plant pathogens, this motif has been described as a common feature of the transcriptional regulation of T3SS genes [97]. In *P*. *syringae*, this motif is recognized by the alternative sigma factor encoded by *hrpL*, whose expression is controlled by two homologous proteins, HrpR and HrpS. Homologs of HrpL and HrpR/HrpS have been found in plant-beneficial *Pseudomonas* strains [64,69] and such a regulation pathway has only been demonstrated in one of these strains, *P*. *marginalis* SBW25 [96]. Interestingly, the expression of *hrpR* and *hrpS* is regulated by the Gac system, a global two-component transduction system ultimately controlling the expression of a vast array of genes involved in bacterial lifestyle changes in *Pseudomonas* spp., especially virulence and plant protection factors [98,99]. However, even in the *P*. *syringae* complex, mostly encompassing plant pathogens, the influence of the Gac system on T3SS regulation fluctuates [94]. Also, the environmental signal (or signals) triggering the Gac system is still unknown, and what has been shown in plant pathogens regarding T3SS regulation may not fully match the actual regulation pathways in plant-beneficial *Pseudomonas* strains. What distinguishes a plant-beneficial strain from a plant-pathogenic one can indeed be based on slight differences in the regulation of gene expression [100].

## 5. Concluding Remarks

**Figure 2:**
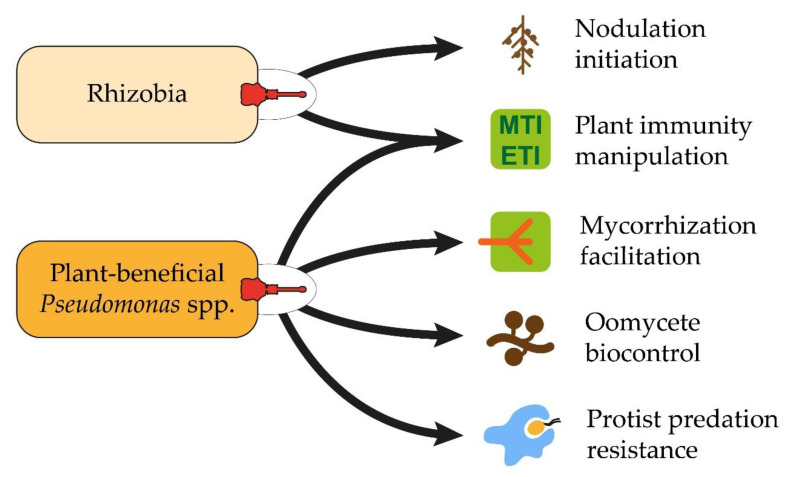
The phytobeneficial roles of T3SSs in rhizobia and *Pseudomonas* spp. MTI: microbe-associated molecular pattern (MAMP)-triggered immunity; ETI: effector-triggered immunity.

Our current knowledge on T3SSs of plant-beneficial bacteria points to the versatile functions they play in interkingdom interactions (Figure 2). While much has been discovered about T3SSs in rhizobia and their role in symbiotic relationships, research on T3SSs in plant-beneficial *Pseudomonas* spp. is lacking. These bacteria are well known for using multiple tools to affect their neighbors. Some of these tools have been extensively studied, such as antibiotics or interference with plant hormone signaling, while others, such as T3SSs, have been under much less scrutiny. Using the little available knowledge about T3SSs in plant-beneficial *Pseudomonas* strains, we hope to have demonstrated that these nanomachines have nonetheless significant impacts in the rhizosphere. They often even play a major role in plant-beneficial effects, by mediating interactions with diverse eukaryotes, not only the plant. We therefore believe that the development of efficient *Pseudomonas*-based biocontrol solutions, which is gaining increasing interest in the context of sustainable farming, would greatly benefit from a deepened understanding of the roles and the underlying mechanisms of T3SSs. A better characterization of additional T3SSs and effectors in rhizobia could also contribute to an improvement in biofertilization solutions. Many research avenues have been opened to achieve this. We should now increase our efforts to better characterize the roles played by these nanomachines and take benefit of their implication in plant-microbe interactions.

## Figures and Tables

**Table 1:** Complete T3SS gene clusters found in plant-beneficial *Pseudomonas* spp.

T3SS Family	*Pseudomonas* Species	Strain(s)	Demonstrated Plant-Beneficial Effect(s)							References
			B	F	O	N	PGP	ISR	MHB	
Hrc1	*P. aridus*	R11-23-07, R2-7-07, R1-43-08	•	•	•					[17]
	*P. brassicacearum*	3Re2-7		•						[59]
	*P. brassicacearum*	LBUM300		•	•					[60]
	*P. brassicacearum*	Q8r1-96		•						[15,61]
	*P. chlororaphis*	ToZa7	•	•	•					[17]
	*P. indica*	PIC105	•	•	•					[62]
	*P. lactis* *	SS101			•					[15]
	*P. orientalis*	R2-66-08W, R4-35-08, L1-3-08, 8B	•	•	•					[17]
	*P. marginalis* *	SBW25			•		•			[15,63]
	*P. paracarnis* *	A506	•							[15]
	*P. paracarnis* *	WCS374		•				•		[16,64]
	*P. simiae*	PCL1751		•			•			[58,65]
	*P. simiae* *	R81					•			[66]
	*P. simiae*	WCS417	•	•	•	•	•	•		[16,64]
	*P. synxantha*	2-79, 30B, LBUM223, R6-28-08	•	•	•					[17]
	*Pseudomonas* sp.	BG33R				•				[15]
Hrc1 (continued)	*Pseudomonas* sp.	KD			•					[67,68]
	*Pseudomonas* sp.	PIC25	•	•	•					[62]
	*Pseudomonas* sp.	PICF141		•	•					[62]
	*Pseudomonas* sp.	Pf29Arp		•						[69]
	*Pseudomonas* sp.	R5-89-07	•	•	•					[17]
SPI-1 (Inv-Mxi-Spa)	*P. chlororaphis*	ATCC 17411, ATCC 17809, ChPhzS140, SLPH10	•	•	•					[17]
	*P. monteilii* *	B001						•		[70,71]
	*Pseudomonas* sp.	GM49							•	[72,73]
	*Pseudomonas* sp.	UW4					•			[74]
SPI-2 (Esc/Ssa)	*P. chlororaphis*	TAMOak81, B25, PA23, DSM 21509	•	•	•					[17]
Hrc1 and SPI-1 (Inv-Mxi-Spa)	*P. aridus*	R2-37-08W, R3-18-08, R4-34-07, R4-39-08, R2-60-08W, R4-35-07, R3-52-08, R2-4-08W, R2-54-08W	•	•	•					[17]
	*“P. ogarae”*	F113		•	•	•				[73]
	*Pseudomonas* sp.	2P24		•						[75]
	*Pseudomonas* sp.	Q2-87		•						[15]
Hrc1 and Rhizobiales	*P. yamanorum*	LBUM636	•	•	•					[17]
	*Pseudomonas* sp.	BBc6R8							•	[76]
	*Pseudomonas* sp.	LBUM920	•	•	•					[17]

* Strain renamed according to digital DNA-DNA hybridization (dDDH) values provided by the Type (Strain) Genome Server [77]. B, F, O and N, biocontrol abilities against pathogenic bacteria, fungi, oomycetes, and plant-parasitic nematodes, respectively; PGP, direct plant-growth promotion; ISR, induction of systemic resistance in plants; MHB, mycorrhiza helper bacterium.

## Data Availability

The GenBank accession numbers (https://www.ncbi.nlm.nih.gov/) for the amino acid sequences used in Figure 1 are provided in brackets (see Figure 1).
